# Effect of neck extension on the advancement of tracheal tubes from the nasal cavity to the oropharynx in nasotracheal intubation: a randomized controlled trial

**DOI:** 10.1186/s12871-019-0831-6

**Published:** 2019-08-17

**Authors:** Hyerim Kim, Jung-Man Lee, Jiwon Lee, Jin-Young Hwang, Jee-Eun Chang, Hyun-Joung No, Dongwook Won, Hyung Sang Row, Seong-Won Min

**Affiliations:** 1 Department of Anesthesiology and Pain Medicine, Seoul Metropolitan Government Seoul National University Boramae Medical Center, 20 Boramae-ro 5-gil, Dongjak-gu, Seoul, 07061 Republic of Korea; 2 Department of Anesthesiology and Pain Medicine, Keimyung University Dongsan Medical Center, Keimyung University School of Medicine, 1095 Dalgubeol-daero, Dalseo-gu, Daegu, 42601 Republic of Korea; 3 Department of Anesthesiology and Pain Medicine, Seoul National University College of Medicine, 101 Daehak-ro, Jongno-gu, Seoul, 03080 Republic of Korea; 4 Department of Anesthesiology and Pain Medicine, Anesthesia and Pain Research Institute, Yonsei University College of Medicine, 50-1 Yonsei-ro, Seodaemun-gu, Seoul, 03722 Republic of Korea; 5 Department of Anesthesiology and Pain Medicine, Seoul National University Hospital, 101 Daehak-ro, Jongno-gu, Seoul, 03080 Republic of Korea

**Keywords:** Intubation, Nasotracheal, Neck extension, Tracheal tube

## Abstract

**Background:**

Clinicians sometimes encounter resistance in advancing a tracheal tube, which is inserted via a nostril, from the nasal cavity into the oropharynx during nasotracheal intubation. The purpose of this study was to investigate the effect of neck extension on the advancement of tracheal tubes from the nasal cavity into the oropharynx during nasotracheal intubation.

**Methods:**

Patients were randomized to the ‘neck extension group (E group)’ or ‘neutral position group (N group)’ for this randomized controlled trial. After induction of anesthesia, a nasal RAE tube was inserted via a nostril. For the E group, an anesthesiologist advanced the tube from the nasal cavity into the oropharynx with the patient’s neck extended. For the N group, an anesthesiologist advanced the tube without neck extension. If the tube was successfully advanced into the oropharynx within two attempts by the same maneuver according to the assigned group, the case was defined as ‘success.’ We compared the success rate of tube advancement between the two groups.

**Results:**

Thirty-two patients in the E group and 33 in the N group completed the trial. The success rate of tube passage during the first two attempts was significantly higher in the E group than in the N group (93.8% vs. 60.6%; odds ratio = 9.75, 95% CI = [1.98, 47.94], *p* = 0.002).

**Conclusion:**

Neck extension during tube advancement from the nasal cavity to the oropharynx before laryngoscopy could be helpful in nasotracheal intubation.

**Trial registration:**

ClinicalTrials.gov Identifier NCT03377114, registered on 13 December 2017.

## Background

Nasotracheal intubation is useful in some clinical situations, such as oral and maxillofacial surgery. Anesthesiologists sometimes encounter resistance in the advancement of a tracheal tube inserted via a nostril from the nasal cavity to the oropharynx before introducing a laryngoscope during nasotracheal intubation. This resistance might be caused by a large-sized tracheal tube compared to the nasal cavity [1] or blockage by the posterior wall of the nasopharynx. Clinicians can easily detect the former as a cause of resistance and resolve the problem by changing to a smaller tube. Regarding the latter cause, the blockage might be possibly due to that the angle between the nasal floor and posterior wall of the nasopharynx is about 90 degrees.

Previous review articles on nasotracheal intubation [2–4] have not addressed the role of neck extension in tracheal tube advancement from the nasal cavity to the oropharynx. A few previous articles introduced resistance in tube advancement from naso/oro-pharyngeal junctional space, and authors commented rotation of the tracheal tube inserted in the nasal cavity could help tube passage at the posterior nasopharynx [2, 3]. However, it has been not investigated yet. It is well known that neck extension is useful in laryngoscopy during tracheal intubation [5, 6]. However, this maneuver seems to be not well-acknowledged to most clinicians for tube advancement from the nasal cavity to the oropharynx in nasotracheal intubation.

Some previous studies presented that red rubber catheters or nasogastric tubes were helpful for safer nasotracheal intubation [7–9]. Even though these materials can help successful advancement of tracheal tubes from the nasal cavity to the oropharynx before laryngoscopy, the aid of them may need additional cost, time, and experienced assistants. If any method with significant efficiency for the advancement of tracheal tubes from the nasal cavity to the oropharynx will be introduced, that will be meaningful.

The aim of this study was to assess the effect of neck extension during the advancement of a tracheal tube from the nasal cavity to the oropharynx on the success of tube advancement. The primary hypothesis of this study was that neck extension could assist in the successful advancement of a tracheal tube from the nasal cavity to the oropharynx in nasotracheal intubation.

## Methods

This prospective, randomized controlled study was approved by the institutional review board of the Seoul Metropolitan Government Seoul National University Boramae Medical Center (no: 16–2017-64), and written informed consent was obtained from all subjects. The trial was registered prior to patient enrollment at ClinicalTrials.gov (NCT03377114). This manuscript adheres to the applicable 2010 CONsolidated Standards of Reporting Trials guidelines. American Society of Anesthesiologists (ASA) physical status I-II adult patients (≥ 18 years old) requiring nasotracheal intubation were recruited between December 2017 and June 2018. Patients with cervical spine instability, coagulopathy, history of taking an anticoagulant, or those in need of awake intubation were excluded from this study.

Patients were randomly assigned to the neck extension group or the neutral position group with 1:1 ratio. An investigator who did not participate in this study generated the randomization allocation sequence using computer-generated block randomization (4-sized blocks, including letters A and B). Each generated letter was concealed in a sequentially numbered opaque envelope. Enrolled patients were allocated to the assigned groups depending on the letter (A to the neck extension group and B to the neutral position group) inside the envelope, and the concealed envelope was opened in an operating theatre by an assistant nurse on the operating day. We blinded the assigned group to each patient in the trial.

**Fig. 1:**
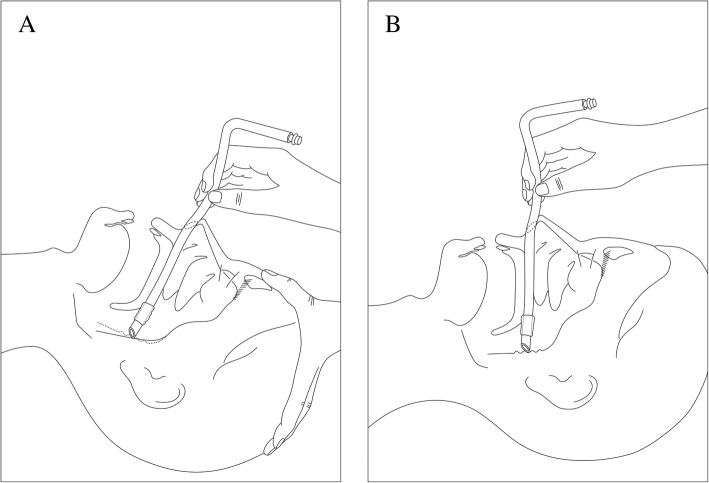
Schematic diagram of two methods with or without neck extension for tube advancement from the nasal cavity into the oropharynx. **a** depicts advancement of a preformed nasal RAE tube with neck extension. With neck extension, the angle between the axis of the distal part of the tube and the posterior wall of the nasopharynx could be obtuse, and the wrinkled soft tissue might be spread, such as a change from dotted lines to solid lines. Based on our results, we hypothesized that these possible changes might aid smooth advancement of the tube. However, these hypotheses were not investigated in the study. **b** depicts advancement of a preformed nasal RAE tube without neck extension (neutral head position). Although not presented in the results, the angle between the nasal floor and the posterior wall of the nasopharynx, without neck extension, was measured as about 100 degrees in the sagittal view of maxillofacial computed tomography of 39 among the study subjects. Also, we observed the angle became widen with neck extension in 3 patients who were preoperatively examined about cervical spine mobility, when we reviewed radiologic findings of cervical spine series of flexion/neutral/extension postures. RAE indicates Ring-Adair-Elwyn

Patients were admitted to the operating theatre without any premedication. Patients were positioned on the operating table in a supine position with a standard pillow under the head. A preformed nasal RAE (Ring-Adair-Elwyn) tube (Mallinckrodt Preformed Nasal RAE tube; Covidien, Mansfield, MA) was softened in warm sterile saline at 45 °C prior to use (inner diameter (ID) 6.5 mm for females, 7.0 mm for males). Pulse oximetry, electrocardiography, and non-invasive arterial blood pressure were monitored in a standard manner. Anesthesia was induced with intravenous administration of glycopyrrolate (0.2 mg), lidocaine (30 mg), propofol (1.5 mg/kg), and fentanyl (100 μg). After confirming that patients became unconscious, patients’ lungs were ventilated by manual bag/mask ventilation with oxygen and sevoflurane after the nares were topically pretreated with sterile cotton swabs soaked with a diluted solution of 0.01% epinephrine. Next, rocuronium (0.6 mg/kg) was administered to achieve muscle relaxation for tracheal intubation. During manual bagging, an investigator measured the distance from the midpoint of the nasal tip to the posterior wall of the nasopharynx using a fiberscope with an outer diameter of 4.1 mm (Olympus LE-P; Olympus Optical Co. Tokyo, Japan) with a brief pause in manual bagging. Immediately prior to nasotracheal intubation, the thermo-softened RAE tube was well lubricated with lidocaine jelly and gently inserted into the nostril that was determined to be most suitable for surgery with the nasal tip lifting maneuver [10]. When the tube was inserted into the nasal cavity approximately 3–4 cm, further advancement of it into the oropharynx was performed as followings in accordance with the assigned group. In the neck extension group, an anesthesiologist advanced the tube into the oropharynx after extending the patient’s neck, as shown in Fig. 1a. Neck extension during tube advancement was performed with a routinely used manner, without any fixed angle, for tracheal intubation in common clinical situations. For patients in the neutral position group, the intubation performer continued to advance the tube to the oropharynx with the patient’s head in a neutral position, as shown in Fig. 1b. During this advancement, the performer and the investigator checked the resistance by blockage at the posterior wall of the nasopharynx. In the case of blockage, the investigator measured the inserted length of the tube at the moment of blockage by using thread as in a previous study [11]. Following this measurement in the case of blockage, we attempted to advance the tube one more with the same maneuver after withdrawing the tube 1–2 cm. If tube advancement succeeded within the two attempts, we recorded the case as ‘success.’ Otherwise, we recorded the case as ‘failure.’ In the case of ‘failure,’ we tried to advance the tube into the oropharynx with alternative methods including change of neck position for tracheal intubation. After finally successful advancement of the tube into the oropharynx, standard nasotracheal intubation was performed using a laryngoscope with the aid of Magill forceps. During this intubation procedure, a second investigator recorded the time from initiation of tube insertion via the nares to passage of the tube into the oropharynx and total intubation time. Individuals who performed tracheal intubation were board-certified anesthesiologists.

After completion of nasotracheal intubation, another investigator checked whether the tube had passed through the upper pathway or the lower pathway with the fiberscope as in a previous study [10]. Also, the investigator checked the presence and grade of epistaxis or nasopharyngeal bleeding. The severity of epistaxis or nasopharyngeal bleeding was classified as “no bleeding,” “blood-tinged mucus,” “mild bleeding,” or “severe bleeding.”

### Statistical analysis

Patient characteristics and outcome measures, including patient age, height, weight, body mass index (BMI), and intubation time are presented as the mean ± standard deviation (SD). Numbers with percentages are presented for sex and the success in advancing a tracheal tube from the nasal cavity to the oropharynx in the first two attempts (the first and second attempts). Additionally, the incidence by which tracheal tubes passed through the lower pathway in the nasal cavity and the incidence of epistaxis or nasopharyngeal bleeding are presented as numbers with percentages.

We compared the success rate in advancing the inserted tube via a nostril from the nasal cavity to the oropharynx in the first two attempts between the two groups (primary outcome) with a χ^2^ test. We compared the incidence of nasal bleeding between two groups with a χ^2^ test. We assessed the incidence of tube passing pathway to verify the results of our previous study [10]. We also assessed the intubation times between two groups with Student’s t-test. The odds ratio or mean difference was calculated for appropriate outcomes. A *p*-value < 0.05 was considered statistically significant. Statistical analyses were performed using SPSS Statistics 21.0 software (IBM Corporation, Chicago, IL, USA).

During a literature search, we could not find any previous study investigating the subject of our study. Therefore, we initially planned to perform this trial as a pilot study with a sample size of 66 (33 for each group). We hoped that we would obtain 80% power at the 0.05 significance level to determine that neck extension could help increase the success rate during the first two attempts by 30% compared with a neutral head position. If this goal was reached in this pilot study, we planned to represent the results as the final results of the study on this issue. Alternatively, we planned to perform an additional study with an appropriately calculated sample size on the basis of the results of the present study.

## Results

**Fig. 2:**
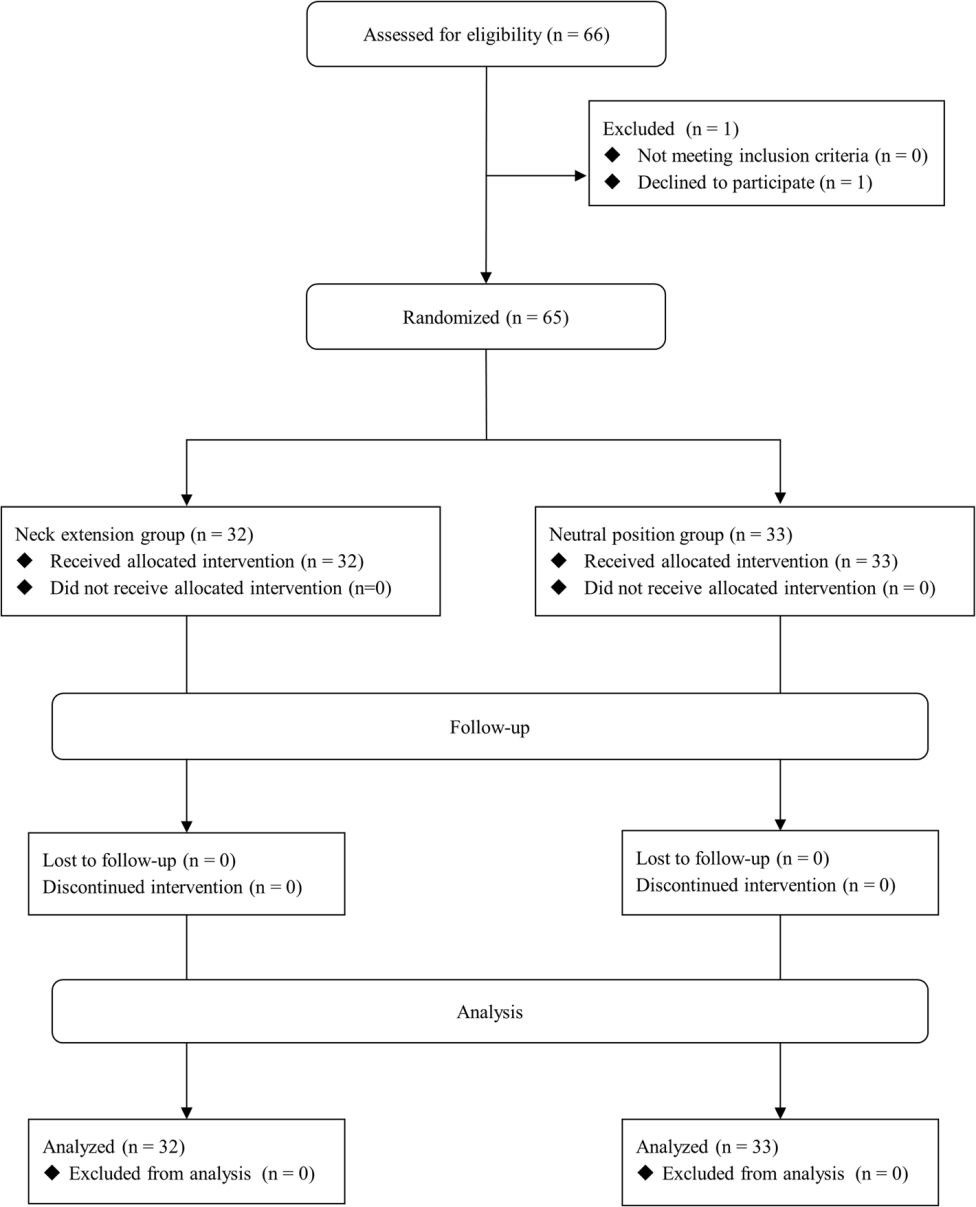
Flow diagram of the study

Patient screening, enrollment, randomization, and analysis are shown in the CONSORT flow diagram in Fig. 2. Sixty-six patients requiring nasotracheal intubation for general anesthesia were enrolled in the study. Patients were randomly assigned to the two groups with a 1:1 ratio. Sixty-five patients completed the present study. One patient in the E group declined participation of the study after the assignment of a group. The demographic data of all patients, who completed the study in both groups, are presented in Table 1. Tracheal intubation was finally successful in all participants. There was no important harm or unintended effect in all participants.

**Table 1:** Patient characteristics

Patient characteristics	Neck extension group (*n* = 32)	Neutral position group (*n* = 33)
Gender (M / F)	17/ 15	21 / 12
Age (y)	42.3 ± 18.2	41.5 ± 18.6
Height (cm)	165.4 ± 10.4	167.2 ± 10.0
Weight (kg)	65.8 ± 13.9	66.5 ± 12.1
BMI (kg/m^2^)	23.8 ± 3.6	23.7 ± 3.5
Nose-posterior wall of nasopharynx distance (cm)	9.6 ± 0.8	9.7 ± 0.8

*BMI* body mass index. Data are presented as the mean ± standard deviation or numbers

The success rate of tube passage in the first two attempts was higher in the E group than in the N group (93.8% vs. 60.6%, odds ratio (OR) = 9.75; 95% confidence interval (CI) = [1.98, 47.94], *p* = 0.002). Additionally, the success rate of smooth advancement of the tube during the first attempt was significantly higher in the E group (87.5%) than in the N group (51.5%) (OR = 6.59, 95% CI = [1.89, 23.01], *p* = 0.003).

The mean insertion time from tube insertion via the nares to the tube passing into the oropharynx was shorter in the E group (10.3 ± 6.6 s) than the N group (16.5 ± 14.8 s) (*p* = 0.035). However, there was no significant difference among patients who were successfully intubated during the first attempt without any blockage between the two groups (9.3 ± 3.9 s in 28 patients of the E group, 8.2 ± 2.3 s in 17 patients of the N group, *p* = 0.720). The total intubation time was not significantly different between the two groups (61.2 ± 35.3 s in the E group, 69.6 ± 37.4 s in the N group, *p* = 0.356). There was no significant difference in the incidence of epistaxis or nasopharyngeal bleeding between the two groups (5/32 in the E group, 11/33 in the N group; OR = 0.40, 95% CI = [0.20, 2.15], *p* = 0.150). All patients who experienced nasal bleeding in both groups exhibited ‘blood-tinged’ mucus (Table 2).

**Table 2:** Primary and secondary outcomes in the two groups

	Neck extension group (*n* = 32)	Neutral position group (*n* = 33)	OR or MD [95% CI]	*P*-value
Primary outcome				
Success rate in tube advancement at the first two attempts	30/32 (93.8%)	20/33 (60.6%)	9.75 [1.98, 47.94]	0.002
Secondary outcomes				
Nasal bleeding				
Incidence, n(%)	5 (15.6%)	11 (33.3%)	0.4 [0.20, 2.15]	0.150
Severity (no/tinged/mild/severe), n	27/ 5 / 0/ 0	22/ 11/ 0/ 0		
Intubation time				
Time from initiation of inserting tube to passing into oropharynx (s)	10.3 ± 6.6	16.5 ± 14.8	−6.2 [− 11.9, − 0.5]	0.035
Total intubation time (s)	61.2 ± 35.3	69.6 ± 37.4	−8.4 [− 26.4, 9.7]	0.356

*OR* odds ratio, *MD* mean difference, *CI* confidence interval

For 20 patients who experienced tube blockage in the first attempt, the discrepancy between the distance from the midpoint of the nares to the posterior wall of the nasopharynx and the length of the inserted part of the tracheal tube, which was measured when the tube was blocked during advancement into the oropharynx, was not different (mean difference = 0.18 ± 0.48 cm, 95% CI = [− 0.41, 0.46]) (*p* = 0.111).

Tracheal tubes passed through the lower pathway of the nasal cavity in 47 patients when we inserted the tube via a nostril with nasal tip lifted in all patients of both groups (72.3, 95% CI = [61.3, 83.3%]) (Table 3).

**Table 3:** The incidence of tracheal tube passage through the lower pathway in the nasal cavity

	Neck extension group (*n* = 32)	Neutral position group (*n* = 33)	Total (*n* = 65)
Lower pathway, n(%) [95% CI]	22 (68.8%)	25 (75.8%)	47 (72.3%) [61.3, 83.3%]

Lower pathway indicates the pathway below the inferior turbinate and above the nasal floor in the nasal cavity. *CI* confidence interval

## Discussion

Our study demonstrated that neck extension during advancing a tracheal tube from the nasal cavity into the oropharyngeal space could assist in smooth passage of the tube. For successful nasotracheal intubation, some previous studies have focused on tube impingement and solutions [12–15]. However, these previous studies mentioned impingement at the hypopharyngeal and laryngeal space but not at the naso/oro-pharyngeal space in fiberoptic intubation. For example, the tube can be impinged at the arytenoid cartilage, vocal cord, epiglottis, or esophageal inlet in fiberoptic nasotracheal intubation, which can be solved by counter-clockwise tube rotation after withdrawal the tube 2–3 cm [14]. Also, if the block occurs due to small-sized nostril before advancing the tube, clinicians can easily realize and solve the problem with changing the nostril side or tube size. Even though there are not these two situations, clinicians commonly encounter resistance in the process of advancing the tube, when it reaches at the posterior wall of the nasopharynx [3]. Our study presented the impingement of the tube in the naso/oro-pharyngeal space and the solution for this issue. Our results showed that the straight distance from the midpoint of the nares to the posterior wall of nasopharynx was very similar to the inserted tube length when the tube was blocked during advancement.

We hypothesized that the angle between the posterior wall of the nasopharynx and nasal floor was about 90 degrees. Although we could not find out any reference about the angle, the mean of the angle and SD was 100.3 ± 7.8 degree when we measured the angle in a sagittal view of preoperative computed tomography images of 39 subjects among all participants in our study. Wrinkles in the posterior nasopharyngeal wall might be a possible cause of blockage because the wall is covered by lymphoid tissue that often undergoes hypertrophy (adenoid) during the transition period to puberty [16]. There are some folds such as salpingopharyngeal fold, salpingopalatine fold, or torus tubarius [17].

For blockage in tube passing from the nasal cavity to the oropharynx, clinicians usually try re-advancing 2–3 times, which can increase the possibility of nasal bleeding. In extreme cases, the tube might perforate the posterior wall [18–22]. Therefore, some experienced clinicians gently rotate the shaft of the inserted tube in the nasal cavity or extend the patient’s neck while advancing the tube like as our study protocol.

We supposed that neck extension could lead to the traction of naso/oro-pharyngeal soft tissue, as shown in Fig. 1a and b. That is, we hypothesized that the soft tissue could be tightened from Fig. 1b to Fig. 1a, which could make the angle between the posterior wall of the nasopharynx and nasal floor more obtuse than neutral position (about 100 degrees in 39 subjects of our study). Finally, this extension can force the tube tip to slide more smoothly across the surface of the posterior wall of the nasopharynx toward the oral cavity. Additionally, we hypothesized that these series of processes could help in spreading the wrinkles of the posterior pharyngeal wall, which can lead to smooth passage of the tube.

However, these hypotheses were not verified in our study. Nevertheless, we identified that the neck extension could increase the angle between the nasal floor and the posterior wall of the pharynx when we observe the cervical spine lateral view with the patient’s neck flexed/neutral/extended in 3 of our study subjects. The angle changed 103.2–107.8-116.8, 84.6–92.4-102.4, and 89.6–93.8-99.3 degrees respectively in them. Also, we identified that soft tissue such as folds of the posterior nasopharyngeal wall of some patients widened and slightly stretched by neck extension when we observed the posterior wall of the nasopharynx with otolaryngologists using a rigid endoscope in clinical situation of endoscopic sinus surgeries.

Neck extension can lead to the alignment of the three axes, including the oral axis, pharyngeal axis, and tracheal axis [6]. Alignment provides physicians the best view of the glottic opening with a laryngoscope for tracheal intubation. Therefore, neck extension is a very familiar maneuver for clinicians in tracheal intubation. Moreover, this maneuver is very easy to perform and is acceptable for most patients except for those with cervical spine injury [23]. Therefore, this maneuver can easily reduce the spent on nasotracheal intubation and improve patient safety.

We evaluated the tube passing pathway to verify the results of our previous study [10]. Unfortunately, our previous study had a small sample size by mistake. Therefore, it had lower power than originally planned. In the present study, we initially inserted the tracheal tube via a nostril with a nasal tip lifted in all subjects. As a result, 72.3% of tubes passed the lower pathway in the nasal cavity. These results were similar to our previous data (78%) in the nasal tip lifting group [10]. Therefore, the results of our present study supported the results of our previous study.

Tube passage from the nasal cavity to the oropharynx was successfully achieved within the first two attempts in the majority of our study subjects. However, tube passage was attempted four times in 4 patients and five times in 1 patient in our study regardless of the group. We thought that the advancement of the inserted tube should be tried 2–3 times to minimize mucosal injury. According to Lim et al., the Levin tube is useful for guiding a tracheal tube for nasotracheal intubation [9]. Therefore, use of it should be considered after 2–3 times failed tube advancements.

In our study, the time was about 10 s on average for passing the tube into the oropharynx in the neck extension group. Considering a simple process of tube passing into the oropharynx from initiation of tube insertion via a nostril, 10 s may be considered a rather long time. To prevent any injury during nasotracheal intubation, clinicians usually perform thermosoftening and local vasoconstriction like as our practice in the study. Also, gentle advancement of the tube through the nasal pathway and naso/oro-pharyngeal junctional space must be important to minimize injury. However, clinicians tend to be tempted to apply a little more force for the advancement of it into the oral cavity during nasotracheal intubation. Additional force may be effective to shorten the required time for tube passing. However, that can cause mucosal injury in some cases. We focused the minimal mucosal injury and emphasized using minimal force to advance the tube in this trial. Therefore, we needed 10 s for tube passing in the extension group. Although the total of 16 patients experienced nasal bleeding, the severity of it was ‘blood-tinged’ for all of them in our study. They did not need any specific treatment for nasal bleeding. Also, gentle force could affect the success rate of tube passing in the first two attempts. If we had used additional force in tube passing, the success rate would have been higher than our results in both groups.

Our study had many limitations. First, our study had a small sample size. During the literature search, we found no previous studies focusing on our hypothesis. Therefore, we initially designed the present study as a pilot study because we could not calculate adequate sample size. However, when we calculated the sample size based on the hypothesized results of this trial, the required sample size was 64 (32 for each group) with 80% power at the 0.05 significance level. We assumed that the success rate for smooth tube passage into the oropharynx in the first two attempts would be increased by 30% with neck extension compared with 60% with the neutral neck position based on our data (60.6%). Therefore, according to our sample size calculation, we decided that the trial should be terminated without increasing the sample size. Second, our study did not overcome the influence of confounding covariates from personal difference in terms of anatomy. If we performed this randomized controlled study with larger sample size or planned a randomized crossover design study for our interest, we could have minimized the confounding effect. However, we did this study with small sample size, and we could not perform a crossover design study due to ethical reason. If we designed this study with a crossover manner, we had to retry to pass the tube with an alternative method (neck extension or neutral) for a patient after pulling back the tube even though the tube passed successfully into the oropharynx with the first maneuver. Third, we could not thoroughly blind the study protocol to intubation performers because it was difficult to blind anesthesiologists for our study design, including tracheal intubation. Although the outcomes of this study such as success rate were objective variables, there still might be bias from that. However, we believed that intubation performers tried to do their best to pass the tube smoothly from the nasal cavity to oropharynx in all cases of the two groups. Finally, we could not found the exact reason why the neck extension could be helpful for smooth advancing the tube from the nasal cavity to the oropharynx. We just conjectured the angle could become slightly widen by neck extension from cervical spine radiologic series of only three subjects. And, we just observed soft tissue of the posterior nasopharyngeal wall became widen and slightly stretched by neck extension in some patients. Therefore, further study should be necessary to investigate our hypotheses.

## Conclusions

Neck extension during tube advancement from the nasal cavity to the oropharynx may facilitate the tube advancement in nasotracheal intubation. We suggest that this maneuver should be standard for tube advancement for nasotracheal intubation.

## Data Availability

The datasets used and/or analyzed during the current study are available from the corresponding author on reasonable request.

## Abbreviations

ASA: American Society of Anesthesiologists
BMI: body mass index
ID: Inner diameter
OR: Odds ratio
RAE: Ring-Adair-Elwyn
SD: Standard deviation
